# Frequency Fitting Optimization Using Evolutionary Algorithm in Cochlear Implant Users with Bimodal Binaural Hearing

**DOI:** 10.3390/brainsci12020253

**Published:** 2022-02-11

**Authors:** Alexis Saadoun, Antoine Schein, Vincent Péan, Pierrick Legrand, Ludwig Serge Aho Glélé, Alexis Bozorg Grayeli

**Affiliations:** 1 Department of Otolaryngology—Head and Neck Surgery, Dijon University Hospital, 21000 Dijon, France; alexissaadoun.chir@gmail.com (A.S.); antoine.schein@chu-dijon.fr (A.S.); 2 Clinical Support Department, MED-EL, 75012 Paris, France; vincent.pean@medel.com; 3 Institute of Mathematics of Bordeaux, UMR CNRS 5251, ASTRAL Team, Inria Bordeaux Sud-Ouest, University of Bordeaux, 33405 Talence, France; pierrick.legrand@u-bordeaux.fr; 4 Department of Hospital Epidemiology and Infection Control, Dijon University Hospital, 21000 Dijon, France; ludwig.aho@chu-dijon.fr; 5 ImVia Research Laboratory, Bourgogne-Franche Comté University, 21000 Dijon, France

**Keywords:** cochlear implant, binaural hearing, speech discrimination in noise, quality of life, evolutionary algorithm, fitting

## Abstract

Optimizing hearing in patients with a unilateral cochlear implant (CI) and contralateral acoustic hearing is a challenge. Evolutionary algorithms (EA) can explore a large set of potential solutions in a stochastic manner to approach the optimum of a minimization problem. The objective of this study was to develop and evaluate an EA-based protocol to modify the default frequency settings of a MAP (fMAP) of the CI in patients with bimodal hearing. Methods: This monocentric prospective study included 27 adult CI users (with post-lingual deafness and contralateral functional hearing). A fitting program based on EA was developed to approach the best fMAP. Generated fMAPs were tested by speech recognition (word recognition score, WRS) in noise and free-field-like conditions. By combining these first fMAPs and adding some random changes, a total of 13 fMAPs over 3 generations were produced. Participants were evaluated before and 45 to 60 days after the fitting by WRS in noise and questionnaires on global sound quality and music perception in bimodal binaural conditions. Results: WRS in noise improved with the EA-based fitting in comparison to the default fMAP (41.67 ± 9.70% versus 64.63 ± 16.34%, respectively, *p* = 0.0001, signed-rank test). The global sound quality and music perception were also improved, as judged by ratings on questionnaires and scales. Finally, most patients chose to keep the new fitting definitively. Conclusions: By modifying the default fMAPs, the EA improved the speech discrimination in noise and the sound quality in bimodal binaural conditions.

## 1. Introduction

Stereophony is based on combining information in the brain from the two ears. The brain makes use of many different cues to determine the 3D characteristics of an auditory landscape [1]. Their complete combination is required for stereophony to be achieved, but access to only some bilateral cues may still generate substantial benefits.

Advantages of binaural stimulation, as opposed to monaural hearing, are (1) redundancy (summation effect) which enhances the signal detection; (2) localization of the sound-source (in the horizontal plane) based on inter-aural time differences (ITD) and level differences (ILD); and (3) improved speech discrimination in noise when signal and noise are spatially separated (squelch effect).

Moreover, with binaural hearing, the head-shadow increases the signal-to-noise ratio at the farthest ear from the noise (as the head attenuates the noise), while this ratio decreases at the nearest ear to the noise source.

Bimodal binaural hearing refers to the use of a cochlear implant (CI) in one ear in combination with a functional acoustic hearing with or without a hearing aid (HA) on the contralateral side. This association provides adults and children with improved speech perception in quiet and in noise, better music perception, auditory comfort, higher sound quality, enhanced sound localization, and as a result, a better quality of life in comparison to unilateral CI [2,3,4,5]. The improvements are related to the integration of the electric hearing, offering auditory information in a relatively broad frequency range (between 0.07 and 8.5 kHz depending on the brand), and the contralateral acoustic input offering the acoustic fine-structure cues. In addition, bimodal hearing reduces the head-shadow effect due to single-sided deafness (SSD) and restores the binaural squelch and summation effects to some extent [3,6].

However, there is great variability in the integration process; while some bimodal users show substantial benefit, others receive little or no advantage.

This variability could be due to the characteristics of the individual listeners (neural survival, current spread, duration of deafness, lack of cortical plasticity), to different processing times between CI and contralateral HA or NH ear [7], to frequency mismatch between the CI and the contralateral HA or NH ear [8], or differences between automatic gain control (AGC) of the CI and the HA [9]. The latter three parameters could be rectifiable via signal processing and/or mapping [10].

Some patients experience even bimodal interference and report better hearing with one of the ears [11,12,13,14,15]. The possibility of this interference is further supported by the observation that in patients with bimodal binaural hearing, the deactivation of apical CI electrodes coding for frequencies perceived by the aided contralateral ear produces a more natural and less metallic sound without reducing the word discrimination in quiet and in noise [16]. In this case, binaural interactions were apparently improved by suppressing the temporal and/or frequency mismatch in the low frequencies at the cost of reduced cochlear implant performance.

Alternatively, binaural interactions could be improved through frequency band adjustments without electrode deactivation [10], or through adjustment of the temporal processing between the CI and the contralateral HA or ear [7,17].

The facility to obtain the bimodal integration appears to influence the auditory outcome [18]. Apart from patient-related factors, such as deprivation duration or a number of functional channels in the implanted ear, device-dependent factors, such as the asynchronous CI and acoustic inputs, different sound preprocessing strategies in the CI and the HA, or loudness and pitch mismatches can affect the speed and the quality of this integration [18].

Based on these findings, an improvement of the spatial CI coding, which relies on the cochlear tonotopy, could theoretically improve the binaural integration. The Greenwood map offers a relatively precise function describing the physiological place-frequency relation in the human cochlea [19]. With CI, the place-frequency function is different from the physiological cochlear tonotopy and a high inter-individual variability exists since the coverage of the cochlear duct by the electrode array is partial and variable. With time and training, a tolerance to the shift between the electric and acoustic stimuli in terms of temporal and spatial coding appears [18]. In many implantees, a perceptual fusion is observed for two sounds with very different pitches presented simultaneously to both ears [20,21]. 

In a standard CI fitting, the default frequency allocations to active electrodes are not modified and only the sensation of loudness is adjusted [22,23]. Modifying the frequency allocations may improve speech discrimination and music perception [18,24,25], but this type of fitting would involve too many parameters and is not practiced in routine [26]. Moreover, its effect on bimodal binaural patients has not been reported to our knowledge.

We hypothesized that reallocating the frequency bands in the CI would lead to a better fusion of the central binaural information, an improved hearing in noise, and a higher sound quality in bimodal binaural conditions. Binaural redundancy is one of the binaural advantages that could be addressed with bimodal rehabilitation. Binaural redundancy could increase loudness via binaural summation but could also improve the detection threshold and frequency differences, and as a result, the speech recognition in noise [1]. Adults who use bimodal hearing devices seem to benefit from the binaural redundancy [3,12,27] but not always [28]. An inter-aural mismatch may significantly limit this effect. In NH subjects listening to bilateral CI simulations with varying virtual electrode positions [29], the maximum binaural summation benefit for speech in quiet and in noise was observed when the inter-aural mismatch of the virtual electrode positions was ≤1 mm. 

Moreover, inter-aural mismatch has also been shown to limit speech understanding in noise when signal and noise are spatially separated [10,30]. This is due to distortions of ITD and ILD [31,32]. 

A place-matched frequency mapping based on electrode location could be hampered by the difficulty to determine the amount of neural survival or local electric stimulation interactions in the cochlea for an individual [10,33]. ITD was used to evaluate inter-aural place mismatch [31,33] and the results were close in accordance with CT scan estimates, but the studies were limited to SSD and bilateral CI patients. No study has been carried out with bimodal patients with CI and contralateral HA. 

In the bimodal context, patients wear a HA on the contralateral ear with various signal processing programs (number of channels, frequency bandwidths, etc.). Since the number of fitting combinations is very high, artificial intelligence can be employed to search this vast domain for the best solution.

Evolutionary algorithms (EA) are a family of algorithms inspired by Darwin’s theory of evolution [34,35]. Initial individuals, represented in our case by the set of frequency ranges for all electrodes (fMAPs), are submitted to the constraint of the environment (i.e., audiometric scores). Their adaptation, stochastic mixing of their genes (i.e., frequency band allocations), and the possibility of mutation (stochastic changes in the fMAP) yield offspring (new fMAPs) generation after generation (iterations). Each fMAP is submitted to an evaluation by a speech audiometric test in noise to obtain a score for each fMAP, represented by a word recognition score (WRS) out of ten. Based on this score, the best solutions are selected and combined to obtain more performant fMAPs. These algorithms provide a wide exploration of solutions in a predefined domain that could not otherwise be conducted in a timely fashion, even by an expert [36]. By comparison, the existing deterministic and probabilistic algorithms, such as the one used in the only computer-based fitting aid, the Fitting to Outcome Expert (FOX) system, tend to modify the settings to approach an ideal situation with predefined parameters (T- and M-levels, gains, [37,38]). Moreover, the frequency bands are not considered as a parameter [38].

The objective of the present study was to develop and evaluate a fitting protocol based on the CI frequency reallocation for bimodal binaural CI users with different CI brands using an interactive EA method. 

## 2. Materials and Methods

### 2.1. Participants

Twenty-seven adults (10 men, 17 women) volunteered to participate in this monocentric and prospective study. All participants were unilateral CI users for a minimum of 6 months with functional contralateral hearing (normal hearing or HA). Their mean age was 58 ± 16.7 years (median: 64, range: 20–80 years). The average duration of the deafness before CI was 23.5 ± 15.8 years (median: 19, range: 1–55 years). In the implanted ear, patients wore various brands of CIs and coding strategies. In the contralateral ear, 23 participants wore a behind-the-ear HA which was fitted with a NAL-NL1 protocol and checked by their audiologist within the 3-month period before inclusion. Three participants had contralateral normal hearing (Table 1).

### 2.2. Experimental Setup

At inclusion, the clinical and audiometric data were obtained, and each CI was fitted with an fMAP based on the EA. Other fMAPs already available on the processor were left unmodified. Participants were asked to use the EA-based fMAP as much as possible, but they were free to switch to their usual fMAPs ad lib. The second session was conducted 45 to 60 days later. Patients were again evaluated with a pure-tone, speech recognition test in quiet and noise in free-field-like conditions and questionnaires. The main criterion of the study was the improvement of the word recognition score (WRS) in noise with EA-based fMAP.

### 2.3. Audiometry

All evaluations were performed in the bimodal binaural condition in an audiometry booth with a calibrated audiometer (AC40^®^, Interacoustics, Middelfart, Denmark). The signal was delivered by a loudspeaker (Planet M, Elipson, Champigny, France) placed at the level of the head 1 m in front of the participant. 

French Fournier lists were used for the speech audiometry in this study [44].

At the initial and the final evaluation, the audiometry tests included:

- A pure-tone audiometry in free-field-like condition;
- A speech recognition test in quiet with monosyllabic words providing the WRS in quiet;
- A speech recognition test in noise: both signal and noise (white noise at 60 dB SPL) were delivered by the same loudspeaker;
- In a preliminary trial with different lists of 20 words, the signal-to-noise ratio (SNR) was individually adapted (−7, 0, +5, or +10 dB) to obtain a percentage of WRS between 3/10 and 7/10. Every patient kept its individually adapted SNR at the same level through the follow-up. Two series of words were also administered for the initial and the final evaluations.

During the EA-based fitting, each generated fMAP was tested with a different series of 10 words to obtain a WRS in noise out of ten at the same level of SNR used for the evaluations. 

No feedback was provided during speech recognition tests.

The improvement of the WRS in noise on 20 words at the initial and at the final evaluation was the main judgment criterion of the study.

### 2.4. Questionnaires

We also asked the participants to complete a quality-of-hearing questionnaire, APHAB (Abbreviated Profile of Hearing Aid Benefit) in its French version related to their handicap before and after the new CI fitting [45]. The questionnaire includes 24 questions on different everyday-life situations related to hearing function. They are divided into 4 categories: Ease of Communication (EC), Reverberation (RV), Background Noise (BN, communication in environments with high background noise), and Aversiveness (AV). It provides a global score and 4 subdomain scores. The Hearing Implant Sound Quality Index score (HISQUI19, [46]) comprises 19 questions on the sound quality perceived by the CI. Scores range from 19 (poor) to 133 points (excellent). In addition, a shortened Munich Music questionnaire (MMQ) [47] was administered including categorical ratings of “metallic”, “clear”, “pleasant”, and “natural” qualities of the musical sounds plus the following questions with forced categorical responses: How long do you listen to music since the last CI fitting? (<30 min/30–60 min/60–120 min/ >120 min); Can you distinguish between high and low notes? (Yes/no); Do you normally feed music directly into your speech processor? (Yes/no). Finally, patients rated the global sound quality by a Likert scale (natural sound and voices, auditory comfort in silence and in noise; scores ranging from 1, “not at all” to 5, “totally agree”).

### 2.5. Frequency Reallocation with the Evolutionary Algorithm

Evolutionary algorithms are calculation methods based on biological evolution. Among this family of algorithms, the most popular are genetic algorithms [48,49,50,51,52,53]. In this paper, we propose a hybrid algorithm at the intersection of a genetic algorithm and an evolutionary strategy. Indeed, we will manipulate real values and apply Gaussian mutations derived from evolutionary strategies, but we will also perform locus type crossovers, which are usually used in genetic algorithms. Finally, since the evaluation step is carried out without a mathematical evaluation function, but only based on the hearing test results, we consider this approach as an interactive evolutionary algorithm.

The general idea is to bring changes to a set of solutions in the optics of improving them by gradual changes and the assessment of the effects of these changes. During this process, the initial values can be changed randomly in a predefined range, especially to generate the initial set of solutions (parents). Later, limited random changes are also introduced in the process to create new solutions (crossover and mutations). This characteristic classes the evolutionary algorithms as stochastic [54]. This algorithm employs alternatively, the mathematical operators of initiation, evaluation, variation (by combination and mutation), selection, and replacement. Solutions (fMAPs in our study) are generated by combining the parent solutions based on their performance (speech recognition test in noise). Mathematically, the algorithm attempts to find the shortest way to the highest performance. It considers the previous combinations and their results to propose new solutions. 

**Figure 1:**
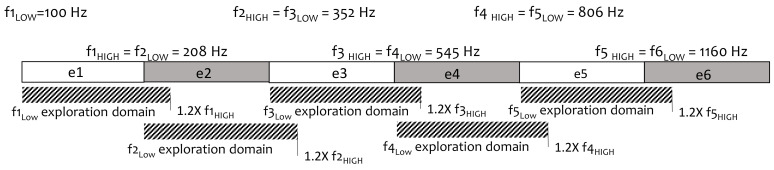
Frequency MAP fitting by an evolutionary algorithm (EA). An example with 5 electrodes (e1 to e5) for an MED-EL device is presented. The initial frequency intervals (f_LOW_ and f_HIGH_ in Hz) and the exploration domains for f_HIGH_ (upper limit of each frequency band) by the algorithm are shown. The f_LOW_ was set equal to f_HIGH_ of the previous electrode to avoid discontinuity and overlap.

**Figure 2:**
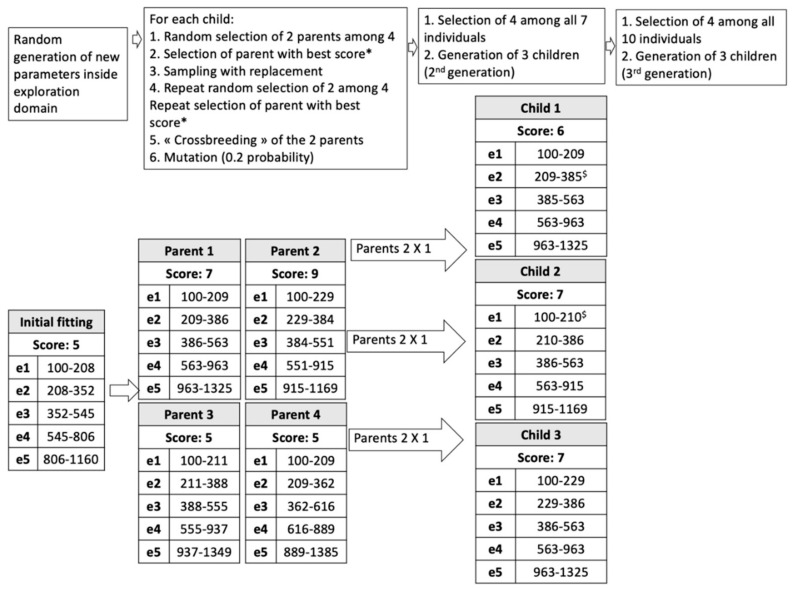
The process of parent and children generation is shown through an example of an MED-EL CI with 5 electrodes. Scores are obtained by speech recognition in noise in a binaural free-field-like condition. * In the case of a tie, the first selected individual is retained (f_HIGH_ mutation $).

The general procedure was as follows (Figure 1 and Figure 2):

1. Input default settings to define the boundaries of the exploration space: exploration domain for each band was set at the lower limit of the same band (f_LOW_) to 1.2 times the upper limit (1.2 × f_HIGH_);
2. Random generation of an initial population in the range allowed for each electrode: 4 parents (i.e., 4 fMAPs P1, P2, P3, P4);
3. Evaluation of P1 to P4 by speech recognition in noise. Each fMAP obtains a score: SP1, SP2, SP3, SP4;
4. Input SP1 to 4;
5. Evolutionary loop, until stop-criteria (number of generations = 3 in our study):
  - Generation of children (3 individuals, first loop: C1, C2, C3);
  - Selection of 2 individuals among the previous generation by tournament: Two individuals of the previous generation are randomly selected; the one with the highest probability is chosen. The previous process is repeated. In this way, two individuals are finally selected;
  - Crossover: combining electrode settings from 2 parent fMAPs to obtain a child;
  - Gaussian mutation: mutations can be applied to f_LOW and_ f_HIGH_ with a probability Pm in the predefined range;
  - Evaluation of the 3 children by WRS yielding scores SCn (first loop: SC1, SC2, SC3);
  - Input SCn;
  - Selection of 4 individuals with the highest WRS among all generated individuals (for the first loop: 7 fMAPS, P1 to P4, and C1 to C3).
6. Output: The best fMAP (highest WRS) obtained during the evolutionary process.

The algorithm was developed using MATLAB (2016a version, MathWorks, Natick, MA, USA) as described before [35]. In this algorithm, each fMAP represented an individual. The default fMAP (factory settings) was used to define the initial frequency bands. Initial fMAPs were generated by EA-based on these initial values. For a new fMAP, the upper limit of each frequency band (f_HIGH_) was determined by the EA in an exploration domain ranging from the lower limit of the same band (f_LOW_) to 1.2 × f_HIGH_ (Figure 1).

Discontinuities and overlaps in the frequency domain were not permitted. Consequently, The f_LOW_ of each frequency band was set equal to the f_HIGH_ of the previous band. Larger values of overlapping and mutation probability would have created a total disruption of the original fMAP, requiring longer adaptation periods, a larger exploration domain with more generations, and longer tests. With these constraints, the default fMAP generated a random initial population of 4 parent fMAPs (Figure 2). 

Each new fMAP was evaluated by WRS in noise (/10) and the result was fed to the algorithm. Two parent MAPs were randomly selected using a tournament selection. The best fMAP (based on WRS) became the first parent and the repetition of the same procedure produced the second parent. These two parents generated a child fMAP by combining their frequency bands (crossover) and applying mutations. During the combination, a variable proportion of the available frequency bands from one parent were combined with those from the second parent to form a new set of frequency bands for the offspring. The combination process did not modify the upper and lower frequency band limits. For the generation of child-fMAPs mutation was applied. 

During a mutation, the upper-frequency band limit was modified in a stochastic manner. This modification was limited to the exploration domain [f_LOW_, 1.2 × f_HIGH_]. The mutation probability for a frequency band in each generated fMAP was set at 0.2, with a standard deviation of 0.1 × frequency band width (Gaussian mutation). The tournament selection was repeated 2 more times to generate 2 other couples of parent MAPs. For these selections, a sampling with replacement strategy was employed. Crossbreeding of each couple produced a child fMAP. Hence, the first generation of 3 child-fMAPs was created and tested by WRS in noise. To create the second generation, 4 parents were selected from 7 already generated fMAPs (4 parents and 3 children). The selection and crossbreeding generated 3 children for the second generation. Finally, for the third generation, 4 parents were selected from 10 generated fMAPs (4 parents and 6 children), and 3 children were obtained. The process was stopped after 3 generations. In total, 13 fMAPs including 4 parents and 9 children over 3 generations, were produced. In the end, the fMAP with the highest WRS was selected. In the case of 2 fMAPs with the same score, the one preferred by the patient based on sound quality was selected. This algorithm differed from the general scheme by the fact that the optimization cycle was halted after 3 generations, and not when an optimization criterion was reached. This specificity was imposed by the length of the procedure and the necessity of multiple speech audiometries, which could not be increased indefinitely.

### 2.6. Fitting Software Programs

BEPS+ research software (Advanced Bionics Research Center, Hannover, Germany) was used for frequency allocation in Advanced Bionics CIs. For all other CIs, routine clinical software was used. The minimal frequency fitting step was 1 Hz for MED-EL and Cochlear CIs, 62 Hz for Advanced Bionics CIs, and 131 Hz for the Oticon Medical CI. For Advanced Bionics and Oticon CIs, the closest frequency increments to the provided fMAP were selected. The minimum frequency band width was 62 Hz for Advanced Bionics and Cochlear CIs, 1 Hz for MED-EL CIs, and 131 Hz for the Oticon Medical CI. These values are obligatory and inherent to the fitting software, the coding strategy, or both.

### 2.7. Determination of Greenwood Frequency MAP in Individual Cochleae

**Figure 3:**
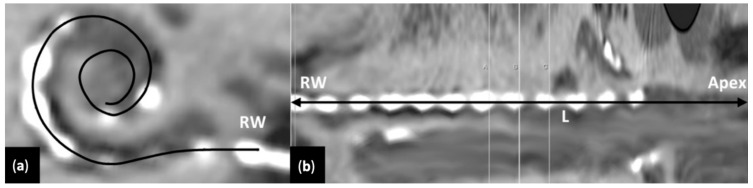
Postoperative CT scans analysis and reconstruction: (**a**) Oblique view of the cochlea on a multiplanar reconstruction (MPR) with minimal intensity projection showed the full length of the electrode array; (**b**) A reconstruction of the image (**a**) in a curvilinear plane unfolded the cochlear spiral; The relative distance of each electrode to the round window (RW) was measured. Since the electrodes are not on the same plane, they cannot be all visualized on the MPR.

The postoperative CT scans were analyzed with Osirix (V4, Pixmeo, Geneva, Switzerland). The length of the cochlea from the round window (RW) to the apex and the position of each electrode from the apex were measured in millimeters (Figure 3). A tridimensional curved multiplanar reconstruction was created. The relative position of each electrode to the apex was expressed as the ratio of the distance between the electrode and the RW to the estimated length of the basilar membrane (in mm). The Greenwood equation was then applied to determine the corresponding tonotopic frequency [19]: F = 165.4 (102.1X − 0.88) where F is the frequency (Hz) and X is the relative distance of the electrode from the round window (distance from round window/entire length of the basilar membrane).

### 2.8. Statistics

Power calculations were carried out by G*Power (v. 1.3.6.9, Heinrich Heine Universität Düsseldorf, Düsseldorf, Germany, [55]). Based on reported studies on speech discrimination in noise in bimodal binaural patients, the inter-individual performance variability was estimated as 20% and a 15% variation of the WRS after EA-based fitting was anticipated. With β = 0.05, and α = 0.05, and for a two-tailed non-parametric paired comparison, 27 participants were required. All patients were included in an intention-to-treat analysis. 

Statistical tests were conducted on Prism, version 8, GraphPad Software, San Diego, CA, USA, 2018. Paired comparisons of continuous variables with non-normal distributions were analyzed by Wilcoxon signed-rank test, and the results were expressed as mean ± standard error of mean, median, and range. ANOVA or mixed-model analysis were employed to compare center frequencies deduced from EA to those obtained from the Greenwood MAPs and the manufacturer’s default settings. Their normal distribution was verified by D’Agostino and Pearson’s test. The results were expressed as mean ± standard error of mean. A *p*-value < 0.05 was considered significant.

## 3. Results

All 27 participants performed the first and the second evaluation sessions and fully completed all evaluation steps. The average duration of the fitting session was 135 ± 30 min. Subjects 2 and 4 (Table 1) did not accept to finish the fitting due to the duration of the test: subject 2 completed the procedure up to the first fMAP of the second generation (8 fMAPs in total) and subject 4 completed the second fMAP of the first generation (6 fMAPs). In patients with MED-EL CIs, the most basal electrodes were deactivated because of a high impedance (patient 1 and 2: electrodes #12, patient 16: electrodes #11 and #12) or a vestibular response (patient 5: electrode #12). For subject 6 (Oticon Medical), the 3 most apical electrodes were deactivated during the first postoperative months because of unpleasant sounds. For the patient with Cochlear CIs, electrode #15 was deactivated because of high impedance (patient 10), and electrodes #1-5 were deactivated because of a vestibular response or no response (Patient 27). The fMAP proposed by the EA excluded the deactivated electrodes as a precondition.

### 3.1. Frequency Band Adjustments with Evolutionary Algorithm

**Figure 4:**
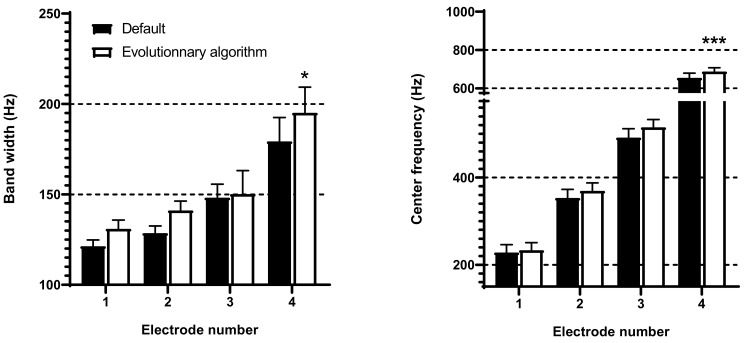
Effect of evolutionary algorithm (EA) after mutations and evolutions on frequency bandwidth, and center frequencies for the 4 most apical electrodes (electrode 1 representing the most apical as in MED-EL and Advanced Bionics CIs). Values are expressed as mean ± standard error of mean (*n* = 27). * *p* < 0.05 for the effect of EA on band width, and *** *p* < 0.001 for the effect of EA on center frequencies; in both analyses, *p* < 0.001 for the effect of electrode number and no significant interaction, two-way ANOVA.

The algorithm suggested a different frequency allocation per electrode than the default setting. The EA yielded an enlargement of the bands in the low frequencies (4 most apical electrodes, Figure 3). Moreover, the EA shifted the center frequencies (Fc) of these apical electrodes toward higher values regardless of CI brand or the number of available electrodes (Figure 4).

In patients with Cochlear or Advanced Bionics CIs, several frequency bands were dramatically narrowed while the bands allocated to the neighboring electrodes were significantly widened, suggesting the detection and the resolution of channel interferences (e.g., electrode 16 in patient 3, Appendix A).

**Figure 5:**
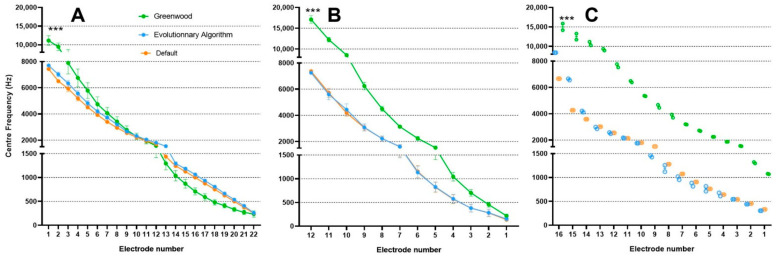
Center frequencies of electrodes according to the Greenwood map calculated on the postoperative CT scan, the default setting, and the evolutionary algorithm (EA) in Cochlear ^®^ (**A**, *n* = 7), MED-EL ^®^ (**B**, *n* = 6) and Advanced bionics ^®^ (**C**, *n* = 2) cochlear implants. Values are expressed as mean ± standard error of mean. For Advanced Bionic (panel **C**), individual values are depicted. Center frequencies according to the Greenwood map differed from default and EA settings. *** *p* < 0.001 for the effects of settings, electrode position, and interaction in all 3 brands, two-way ANOVA.

A postoperative CT scan was available for 15 patients (6 MED-EL, 2 Advanced Bionics, and 7 Cochlear). The center frequencies deduced from the Greenwood map were compared to those from the EA and the default fMAPs (Figure 5, Appendix B). Both EA-based and default fMAPs yielded lower center frequencies than the Greenwood map.

### 3.2. Audiometry

The best fMAP was not always obtained at the last generation (Table 2). In 6 patients, the first generation of fMAPs (parents), which were generated randomly in the predefined domain, yielded the best results. In the remaining cases, the best fMAP was among the first (*n* = 7), the second (*n* = 6), or the third (*n* = 8) generations. In 9 patients, several fMAPs yielded the same optimal WRS (patients 2–4, 8, 9, 11, 20, 21, 24). In these cases, the patient chose the fMAP among those with the highest WRS based on subjective quality of sound.

At the final evaluation, WRS in noise was significantly improved with EA (4.17 ± 0.97, median: 3.5, range: 2–5, with the default fMAP versus 6.46 ± 1.63, median: 7, range: 2–9 with the EA-based fMAP, *n* = 27, *p* = 0.0001, Wilcoxon sign-ranked test). The duration of hearing deprivation was not correlated to the initial or the final WRS (linear regression test, not significant, data not shown). At the final evaluation, the WRS in quiet remained unchanged (9.04 ± 2.01 initially, median: 10, range: 2–10 versus 9.22 ± 1.76, median 10, range 3–10 at the final test, *n* = 27, non-significant, Wilcoxon sign-ranked test). Similarly, the EA-based fitting did not alter the pure-tone average in the free-field-like condition (20.9 ± 9.4 dB HL, median: 20, range: 1.3–47.5, for the initial fitting versus 19 ± 7.2 dB, median 20, range: 1.3–32.5 at the final test, *n* = 27, non-significant, Wilcoxon sign-ranked test).

### 3.3. APHAB Questionnaire

**Figure 6:**
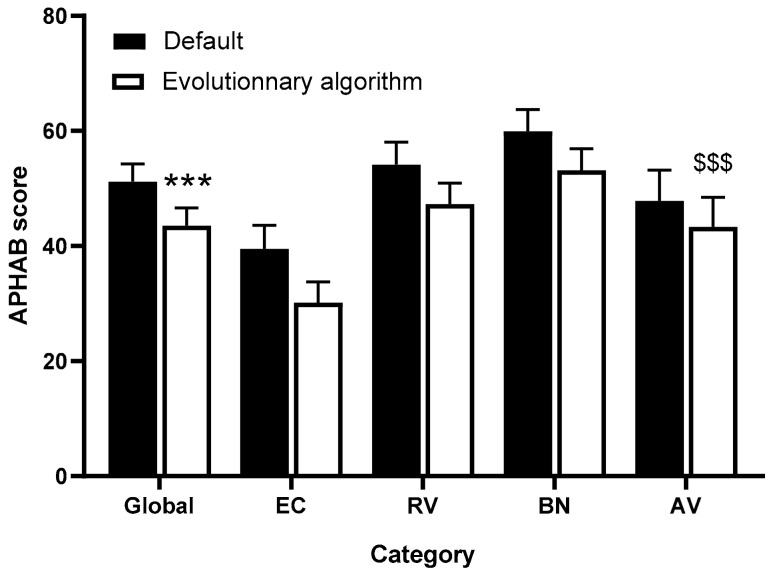
APHAB questionnaire scores with default and evolutionary algorithm settings. Values are expressed as mean ± standard error of mean. *** *p* < 0.001 versus default for the global score, Wilcoxon signed-rank test. $$$ *p* < 0.001 for the effect of setting, and *p* < 0.01 for the effect of APHAB ranges, no significant interaction, 2-way ANOVA. EC = ease of communication, RV = reverberation, BN = background noise, AV = aversiveness.

The quality of hearing, as assessed by APHAB, was significantly improved (50.4 ± 16.6, median: 55.5, range: 24.1–73.2, for the global score before versus 43.5 ± 16.8, median: 46.6, range: 10.2–66.6, after the EA fitting, *n* = 27, *p* = 0.002 Wilcoxon sign-ranked test). The quality of hearing was also significantly improved in EC, RV, and BN APHAB subdomains (Figure 6).

### 3.4. HISQUI Questionnaire

The quality of hearing, as assessed by HISQUI, was significantly improved (72.2 ± 17.7, median: 70, range: 44–116 before versus 77.9 ± 21.5, median: 75, range: 45–121, after the EA fitting, *n* = 27, *p* = 0.034 Wilcoxon sign-ranked test).

### 3.5. Global Evaluation of the Sound Quality and Music Perception

**Figure 7:**
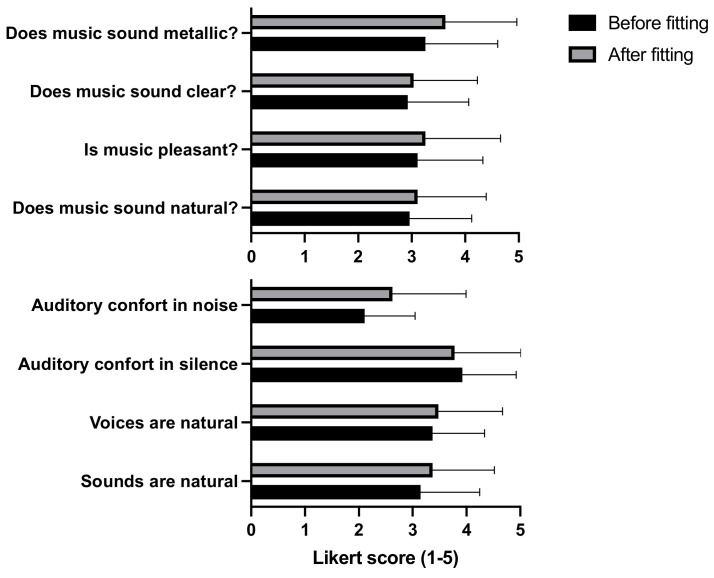
Global evaluation of the sound quality and music perception with default and evolutionary algorithm settings. Values are expressed as mean ± standard error of mean. There is no significant effect of fitting on any item (Wilcoxon signed-rank test).

The music perception quality as evaluated by the MMQ rating, as well as the global sound quality, did not change, as judged by the Likert scale (Figure 7). Where 23 out of 27 participants (85%) preferred the new EA-based fitting to their previous fitting by the expert and chose to keep it after the study.

## 4. Discussion

Optimizing the fitting in CI users with bimodal binaural hearing can be challenging due to a high number of fitting parameters and combinations. We showed that the frequency band distribution by EA improves not only speech discrimination in noise but also the hearing-related quality of hearing, as judged by APHAB and HISQUI questionnaires. The EA-based fitting resulted in a widening of the frequency bands in the low frequencies and a global shift to higher frequencies than those proposed in the default setting, even farther from the original cochlear tonotopy. There were also individual alterations to the default fMAP, presumably depending on specific electrode nerve interactions. Neural survival and current spread could be reflected by these individual alterations because the measures used for the EA were evaluated directly on the patient and were dependent on its extrinsic characteristics [10]. The method was applicable to all different CI brands. 

Speech recognition in noise is probably the most challenging and also one of the most relevant tasks for patients with hearing loss [2]. In case of bimodal or SSD patients, the intersubject variability for binaural results are very large [8]. Wess et al. proposed several possible explanations possible for this variability: (1) intrisinc characteristics of the individual listeners (neural survival, current spread, duration of deafness, lack of cortical plasticity) that cannot be addressed through signal processing; (2) extrinsic distorsions rectifiable via signal processing and/or mapping procedures including different processing times between CI and contralateral HA or NH ear and frequency mismatch between the CI and the contralateral HA or NH ear [10]. For example Zirn et al. [56] and Angermeier et al. [17] showed that sound Localization in Bimodal Listeners could be improved instantaneously when the device delay mismatch (between CI and contralateral HA) was reduced.

Binaural interaction in noisy conditions has been studied by simulating a CI in single-sided deafness [57]. This was obtained by delivering a vocoded speech with variable degrees of mismatch in one ear of eight normal-hearing individuals and evaluating the speech audiometry in noise. The authors show that binaural performances are similar or significantly better than the normal-hearing ear in all cases. Furthermore, in challenging conditions (speech-shaped noise) where the normal ear performance is constrained to the level of the CI performance, a frequency mismatch further degrades the performances, probably by disrupting the binaural interactions. These observations suggest that, in patients with bimodal hearing, reducing the pitch perception mismatch between the CI and the acoustic inputs might enhance hearing in noisy conditions [57].

Even in patients with single-sided deafness (SSD), the contribution of the CI to auditory performances is significant [58,59]. CI decreases the head-shadow effect [58]; increases speech understanding in noise, even in the S0N0 condition (frontal signal and noise) [58,60]; enhances the sound-source localization [58,61]; and improves the patient-reported outcome [62]. In our patients with SSD (patient number 5, 16, and 21), optimization also showed a significant improvement in speech discrimination, except for patient 21 who still felt a subjective improvement with EA fMAP and thus, finally chose it. In line with previous reports, this observation supports the idea that optimized binaural interactions increase performance even with one normal ear.

The idea behind the change in the frequency bands was to optimize the correspondence between the ears and the binaural hearing. Testing these patients in a monaural condition would have probably provided additional interesting data. By reducing channel interactions or by a better correspondence between frequency allocations and functional channels, the EA-based fMAP could also improve monaural hearing.

In a theoretical approach, many have attempted to address the issue of binaural optimization by restoring the pitch-place function of the implanted cochlea according to the original cochlear tonotopy [23,63]. But by looking closer at this problem, the location of the spiral ganglion may be more relevant to the CI stimulation, and its distribution map follows a distinct function from the Greenwood map [63,64]. Nevertheless, attempts to optimize the binaural hearing through frequency allocation according to either the Greenwood or the spiral ganglion map have yielded poor results in general, despite a few individual positive effects on speech discrimination in noise [24,63,65].

Several explanations can be advanced for this failure. The electrode array covers, at best, partially the cochlear apex coding for the low frequencies. Consequently, adjusting the fMAP to the Greenwood function means neglecting a significant part of the spectrum in low and mid frequencies [24]. In the case of shallow insertion, a full and slightly compressed spectral distribution seems to provide better results than a truncated fMAP following Greenwood [24]. Another reason is the number of functional channels (i.e., electrodes eliciting a distinctive pitch) in the implanted cochlea. Theoretically, to each electrode and frequency band should correspond a distinctive auditory nerve ending, but this assumption is far from true in many clinical situations, and frequencies allocated to “dead zones” are lost [66]. Moreover, channel interactions in these cases increase signal distortions and binaural mismatches [66]. Interestingly, the EA-based fitting program indicated an enlargement of frequency bands allocated to several electrodes in our series. We hypothesize that by minimizing the frequency allocation to the electrodes which do not stimulate a distinct neural population, the dissonance decreased, and the hearing improved as it has been already reported [14]. Finally, fitting based on the Greenwood map does not improve the binaural fusion in comparison to the default CI settings since complex central processing adaptations seem to modify the binaural interactions. In patients with bimodal binaural hearing or bilateral CI, two notes separated as far as three or four octaves presented simultaneously to both ears can be perceived with a similar pitch [20,21]. The extent of these alterations depends on many interconnected factors, such as ipsi- and contralateral auditory performances (i.e., speech discrimination, pitch resolution) and the hearing deprivation period [20,21]. 

In contrast to the theoretical approaches based on the Greenwood map, frequency band adjustments have been also tackled through a purely empirical approach [67,68,69]. By studying the correlations between speech and electrode discrimination abilities, several authors could show that low-frequency resolution is a significant factor for speech discrimination in quiet and noise [60,62]. Allocating most of the electrodes to low frequencies (9 out of 10 to frequencies < 2.6 kHz) improved only some aspects of hearing (e.g., vowel discrimination, speech in noise) at the expense of other performances, such as consonant discrimination [69]. Modifying this strategy by affecting only three additional electrodes to low frequencies in comparison to the default setting had small and variable effects [68]. EA appeared to be more performant than empirical systematic protocols by exploiting patient interaction at each step.

In line with studies that suggest that low-frequency resolution is determinant in speech discrimination, pitch-matching studies showed that perceived pitches with CI were lower than what was estimated by the place-pitch function in unilateral CI users with a normal contralateral hearing [70,71,72]. Several clinical studies demonstrated that the adaptation of the peripheral and the central tonotopies to the radical changes of frequency mapping after CI are possible in the majority. This adaptation drives the new tonotopy toward the frequency organization imposed by the CI [72,73,74,75]. Tonotopy adjustments can involve the entire cochlea or only a region [73]. Some patients may not adapt or adapt poorly to these modifications [73] and understanding the reasons for this maladaptation remains a challenge. However, recent results on SSD and bilateral CI suggest poor plasticity of the binaural system to mismatch [74].

In quiet, the maximum speech discrimination was not influenced by the EA-based fitting. This can be explained by the ceiling effect (9.04 ± 2.01 initially versus 9.22 ± 1.76 at the final test). The best fMAP was not always obtained at the last generation (Table 2). In six patients, the first generation of fMAPs (parents), which were generated randomly in the predefined domain, yielded the best results. In these cases, the algorithm could not further improve the result probably due to the ceiling effect again. Indeed, these patients were initially selected with contralateral functional hearing and consequently, had high performances in quiet. The other possible reason is that, while in quiet, patients may rely on their better ear, and in noise, improvement of binaural hearing has a measurable impact on the performance [75]. But if we limit the analyses to the patient who had their best fMAP after the parent generation, we still have a significant improvement for WRS in noise (mean difference = 2.86), APHAB and HISQUI scores, and no difference for tests in silence, which means that there is no reason to exclude those patients. Patients included during the second phase of the study also had WRS in noise with both their default fMAP and EA fMAP at six weeks. If we compare those scores, we still get a significant improvement with the EA fMAP, which confirms that there is an advantage of the EA fitting. Since different word lists were used at each test, a higher repetition of speech tests in noise versus only two tests in quiet does not affect its outcome and does not appear as a plausible cause of bias [76].

Although EA-based fitting procedure is long and can only be applied to motivated patients, conventional protocols of binaural pitch-matching are even more time-consuming, and more difficult [72,77]. Indeed, they require prolonged concentration and the ability to compare pitches of electrical and acoustic sound regardless of their timber, texture, and loudness [77,78]. Unlike these tedious tasks, we chose the discrimination of 10 monosyllabic words in noise which was short but relevant to our objective. On one hand, the performance of the algorithm depended on the reliability of the scores, on the other hand, short tests have the disadvantage of lower test-retest reliability [79]. This tradeoff appeared interesting since it produced a significant improvement in the hearing in noise.

Recently, inter-aural place mismatch was evaluated in bilateral CI and SSD patients with unilateral CI [78] using ITD discrimination (simultaneous bilateral stimulation), place-pitch ranking (sequential bilateral stimulation), and physical electrode location estimated by CT scans. The results showed that binaural processing may be optimized by using CT scan information to program the CI frequency allocation but not place-pitch ranking. However, the study was not carried out with bimodal users (CI + HA). Moreover, a place-matched frequency mapping based on electrode location could be limited due to the difficulty to determine neural survival at the site of each electrode or the electric interactions in the cochlea for individual patients [10]. EA is directly based on the speech discrimination in noise, and thus, might exploit its extrinsic characteristics to optimize the fitting.

The relationship between loudness and pitch can also add complexity to the modifications of frequency band allocations: pitch and loudness are both affected by the rate of stimulation [80,81,82]. Modifying the frequency allocation alters the loudness perception in a non-linear and unpredictable manner [82]. We did not control or investigate the loudness alterations induced by the frequency band shifts because adding loudness adjustments to frequency band modifications would have exponentially increased the possible combinations in the algorithm and would have made the protocol inapplicable. This could be a subject of future research and development in EA-based fitting.

In the future, the algorithm could be integrated into the fitting software to accelerate the procedure and improve its acceptability by the patients. Evaluation procedures other than the WRS, such as musical sound categorization tasks, could be evaluated to improve the process.

## 5. Conclusions

By modifying the default fMAP, the evolutionary algorithm increased the word recognition score in noise and improved APHAB and HISQUI scores. Most of the patients (23 out of 27) preferred the modified fMAP and kept it at the end of the study. These improvements were observed despite the heterogeneity of the CI brands and the contralateral ear condition. These results open insights on integrating this type of approach in standard CI fitting. 

## Figures and Tables

**Table 1:** Patient characteristics.

ID#	Etiology	Hearing Deprivation on Implanted Ear (Years)	Implant	Coding	Contralateral Hearing Aid
1	Otosclerosis	55	MED-EL	FS4 [39]	Chili SP7, Oticon
2	Congenital	28	MED-EL	FS4	Legend 1786, Beltone
3	Congenital	38	COCHLEAR	ACE [40]	Naida Q70-SP, Phonak
4	Ménière	17	COCHLEAR	ACE	Cobalt 8+, Rexton
5	Congenital	23	MED-EL	HDCIS [41]	Normal
6	Congenital	43	OTICON	Crystalis XDP [42]	Nitro 7MI SP, Siemens
7	Congenital	34	COCHLEAR	ACE	Ambra SP, Phonak
8	Sudden SNHL	1	AB	HiRes Optima [43]	Insio 5bX, Siemens
9	Idiopathic	16	AB	HiRes Optima	UPSmart988 GN Resound
10	Lobstein’s disease	42	COCHLEAR	ACE	PHONAK Naida Q50 SP
11	Congenital	39	COCHLEAR	ACE	Siemens Signia Orion 2312
12	Sudden SNHL	18	MED-EL	FS4	Widex Moment
13	Congenital	20	COCHLEAR	ACE	Siemens Pure 500
14	Sudden SNHL	30	COCHLEAR	ACE	Phonak Naida V90 UP
15	Sudden SNHL	13	MED-EL	FS4	Starkey Livio 2400
16	Otosclerosis	2	MED-EL	FS4	Normal
17	Otosclerosis	7	COCHLEAR	ACE	Phonak Audeo B50 R
18	Neurofibromatosis type 2	19	MED-EL	FS4	Phonak Naida Q70 SP
19	Chronic otitis media	42	MED-EL	FS4	No hearing aid
20	Perilymphatic fistula	2	COCHLEAR	ACE	Siemens Rexton Strata 2
21	Perilymphatic fistula	9	COCHLEAR	ACE	Normal
22	Chronic otitis media	50	MED-EL	FS4	Siemens Motion XS
23	Congenital	32	COCHLEAR	ACE	Gn Hearing Resound Alera 7
24	Ménière	2	COCHLEAR	ACE	Belton Identity 86D
25	Meningitis	30	MED-EL	FS4	Siemens Signia Pure 312
26	Ménière	17	MED-EL	FS4	Starkey Resound
27	Sudden SNHL	5	COCHLEAR	ACE	Belton Identity 66D

**Table 2:** WRS for each fMAP generated by the evolutionary algorithm (P1–4 and C1–C9). Asterisk indicates the selected final fMAP. Initial and final WRS (45–60 days after fitting) were tested by 20 words and intermediate WRS by 10 words. All were expressed as a score out of ten. All tests were conducted at the same signal/noise ratio (SNR). Minus sign (-): the patient was not willing to test the fMAPs and abandoned the procedure.

Patient Number	Initial WRS	SNR (dB HL)	Parents				1st Generation			2nd Generation			3rd Generation			Final WRS
			P1	P2	P3	P4	C1	C2	C3	C4	C5	C6	C7	C8	C9	
1	4	10	6	8	6	6	8	6	9 *	6	7	7	7	6	7	9
2	5	5	7	6	10 *	9	4	6	10	9	-	-	-	-	-	6.5
3	4	10	6	4	5	5	5	5	6	8 *	7	8	8	8	6	7.5
4	4	0	5	3	5	6	6 *	4	-	-	-	-	-	-	-	6
5	4	−7	4	4	5	8	6	7	6	5	5	9 *	7	6	8	8
6	5	10	0	2	4	5	2	3	4	5	6 *	5	4	0	5	5
7	3	5	4	7	6	7	5	7	9 *	6	8	4	7	6	7	7.5
8	5.5	5	7	5	6	7	4	5	5	7	5	6	7 *	6	6	7.5
9	5	10	4	6	8 *	5	5	6	6	7	8	7	7	6	5	7.5
10	3	10	2	4	2	3	5	4	5	3	5	4	3	4	9 *	2
11	6	10	6	9	7	5	5	6	8	6	8	8	8	9 *	6	8
12	6	−5	5	4	3	5	7	6	7	4	4	7	4	5	8 *	7
13	3	0	6	2	5	5	4	7	6	2	6	7	4	6	8 *	7
14	3	0	4	8 *	6	2	4	3	6	2	4	3	6	5	5	8
15	4	−5	5	2	4	6	8 *	4	3	7	5	4	1	4	4	7
16	4	−10	5	4	1	2	3	4	6 *	4	5	4	2	3	5	7
17	5	−5	3	5	8 *	3	4	4	5	1	3	7	3	3	2	7
18	4	−5	4	6 *	2	5	2	2	3	5	1	5	5	4	5	7
19	3	−5	3	5	4	4	8 *	4	3	5	5	4	3	5	5	6
20	5	−10	1	1	1	1	7	7 *	4	5	5	7	5	6	5	5
21	4	0	2	5	2	2	3	4	4	2	3	2	5	5 *	4	3
22	3	0	7 *	3	5	5	6	5	4	4	4	2	2	6	6	7
23	3	10	4	2	4	2	1	3	7	8 *	2	4	5	1	4	7
24	5	0	3	5	7	3	3	2	4	3	4	6	7 *	3	2	6
25	5	0	4	1	3	4	5	4	3	3	6 *	4	4	1	1	3
26	3	0	4	3	5	4	2	6	6	4	3	2	4	7 *	4	6
27	4	−5	2	3	3	4	5	3	3	6 *	4	1	4	5	5	7

## Appendix A

**Table A1:** Frequency bands adjustments with the evolutionary algorithm. Values are expressed in Hertz, e# (electrode number), BW (Band Width), **f_LOW_** (Lower Limit, Hz), **f****_HIGH_** (Upper Limit, Hz), CF (Center Frequency, Hz). -: deactivated electrodes.

e#	Initial f_LOW_	Initial f_HIGH_	Final f_LOW_	Final f_HIGH_	Initial BW	Final BW	Initial CF	Final CF
	**Patient 1**							
1	100	208	100	195	108	95	154	147.5
2	208	352	195	332	144	137	280	263.5
3	352	545	332	484	193	152	448.5	408
4	545	806	484	655	261	171	675.5	569.5
5	806	1160	655	984	354	329	983	819.5
6	1160	1643	984	1619	483	635	1401.5	1301.5
7	1643	2303	1619	1917	660	298	1973	1768
8	2303	3208	1917	3048	905	1131	2755.5	2482.5
9	3208	4450	3048	4069	1242	1021	3829	3558.5
10	4450	6155	4069	6076	1705	2007	5302.5	5072.5
11	6155	8500	6076	8500	2345	2424	7327.5	7288
12	-	-	-	-	-	-	-	-
	**Patient 2**							
1	100	208	100	193	108	93	154	146.5
2	208	352	193	284	144	91	280	238.5
3	352	545	284	528	193	244	448.5	406
4	545	806	528	795	261	267	675.5	661.5
5	806	1160	795	1085	354	290	983	940
6	1160	1643	1085	1563	483	478	1401.5	1324
7	1643	2303	1563	2184	660	621	1973	1873.5
8	2303	3208	2184	2811	905	627	2755.5	2497.5
9	3208	4450	2811	4143	1242	1332	3829	3477
10	4450	6155	4143	5134	1705	991	5302.5	4638.5
11	6155	8500	5134	8500	2345	3366	7327.5	6817
12	-	-	-	-	-	-	-	-
	**Patient 3**							
1	188	313	188	298	125	110	250.5	243
2	313	438	298	409	125	111	375.5	353.5
3	438	563	409	471	125	62	500.5	440
4	563	688	471	617	125	146	625.5	544
5	688	813	617	722	125	105	750.5	669.5
6	813	938	722	871	125	149	875.5	796.5
7	938	1063	871	1001	125	130	1000.5	936
8	1063	1188	1001	1067	125	66	1125.5	1034
9	1188	1313	1067	1129	125	62	1250.5	1098
10	1313	1563	1129	1462	250	333	1438	1295.5
11	1563	1813	1462	1669	250	207	1688	1565.5
12	1813	2063	1669	1787	250	118	1938	1728
13	2063	2313	1787	1905	250	118	2188	1846
14	2313	2688	1905	2418	375	513	2500.5	2161.5
15	2688	3063	2418	3030	375	612	2875.5	2724
16	3063	3563	3030	3092	500	62	3313	3061
17	3563	4063	3092	3855	500	763	3813	3473.5
18	4063	4688	3855	4608	625	753	4375.5	4231.5
19	4688	5313	4608	5092	625	484	5000.5	4850
20	5313	6063	5092	5959	750	867	5688	5525.5
21	6063	6938	5959	6460	875	501	6500.5	6209.5
22	6938	7938	6460	7938	1000	1478	7438	7199
	**Patient 4**							
1	188	313	220	368	125	148	250.5	294
2	313	438	368	462	125	94	375.5	415
3	438	563	462	605	125	143	500.5	533.5
4	563	688	605	770	125	165	625.5	687.5
5	688	813	770	832	125	62	750.5	801
6	813	938	832	999	125	167	875.5	915.5
7	938	1063	999	1175	125	176	1000.5	1087
8	1063	1188	1175	1268	125	93	1125.5	1221.5
9	1188	1313	1268	1413	125	145	1250.5	1340.5
10	1313	1563	1413	1702	250	289	1438	1557.5
11	1563	1813	1702	2001	250	299	1688	1851.5
12	1813	2063	2001	2063	250	62	1938	2032
13	2063	2313	2063	2390	250	327	2188	2226.5
14	2313	2688	2390	2772	375	382	2500.5	2581
15	2688	3063	2772	3107	375	335	2875.5	2939.5
16	3063	3563	3107	3834	500	727	3313	3470.5
17	3563	4063	3834	4274	500	440	3813	4054
18	4063	4688	4274	5033	625	759	4375.5	4653.5
19	4688	5313	5033	5580	625	547	5000.5	5306.5
20	5313	6063	5580	6532	750	952	5688	6056
21	6063	6938	6532	7734	875	1202	6500.5	7133
22	6938	7938	7734	7938	1000	204	7438	7836
	**Patient 5**							
1	500	647	500	626	147	126	573.5	563
2	647	837	626	777	190	151	742	701.5
3	837	1083	777	973	246	196	960	875
4	1083	1401	973	1166	318	193	1242	1069.5
5	1401	1812	1166	1493	411	327	1606.5	1329.5
6	1812	2345	1493	2109	533	616	2078.5	1801
7	2345	3034	2109	2614	689	505	2689.5	2361.5
8	3034	3925	2614	3337	891	723	3479.5	2975.5
9	3925	5078	3337	4687	1153	1350	4501.5	4012
10	5078	6570	4687	5877	1492	1190	5824	5282
11	6570	8500	5877	8500	1930	2623	7535	7188.5
12	-	-	-	-	-	-	-	-
	**Patient 6**							
1	195	326	195	326	131	131	260.5	260.5
2	326	456	326	456	130	130	391	391
3	456	586	456	586	130	130	521	521
4	586	716	586	716	130	130	651	651
5	716	846	716	846	130	130	781	781
6	846	977	846	977	131	131	911.5	911.5
7	977	1107	977	1107	130	130	1042	1042
8	1107	1367	1107	1237	260	130	1237	1172
9	1367	1758	1237	1628	391	391	1562.5	1432.5
10	1758	2148	1628	2018	390	390	1953	1823
11	2148	2539	2018	2409	391	391	2343.5	2213.5
12	2539	3060	2409	2930	521	521	2799.5	2669.5
13	3060	3711	2930	3581	651	651	3385.5	3255.5
14	3711	4492	3581	4492	781	911	4101.5	4036.5
15	4492	5404	4492	5534	912	1042	4948	5013
16	5404	6185	5534	6576	781	1042	5794.5	6055
17	6185	7357	6576	7617	1172	1041	6771	7096.5
18	-	-	-	-	-	-	-	-
19	-	-	-	-	-	-	-	-
20	-	-	-	-	-	-	-	-
	**Patient 7**							
1	188	313	188	261	125	73	250.5	224.5
2	313	438	261	424	125	163	375.5	342.5
3	438	563	424	555	125	131	500.5	489.5
4	563	688	555	643	125	88	625.5	599
5	688	813	643	754	125	111	750.5	698.5
6	813	938	754	871	125	117	875.5	812.5
7	938	1063	871	933	125	62	1000.5	902
8	1063	1188	933	1095	125	162	1125.5	1014
9	1188	1313	1095	1157	125	62	1250.5	1126
10	1313	1563	1157	1408	250	251	1438	1282.5
11	1563	1813	1408	1470	250	62	1688	1439
12	1813	2063	1470	1667	250	197	1938	1568.5
13	2063	2313	1667	2048	250	381	2188	1857.5
14	2313	2688	2048	2255	375	207	2500.5	2151.5
15	2688	3063	2255	2603	375	348	2875.5	2429
16	3063	3563	2603	3442	500	839	3313	3022.5
17	3563	4063	3442	3634	500	192	3813	3538
18	4063	4688	3634	4079	625	445	4375.5	3856.5
19	4688	5313	4079	5133	625	1054	5000.5	4606
20	5313	6063	5133	5614	750	481	5688	5373.5
21	6063	6938	5614	6266	875	652	6500.5	5940
22	6938	7938	6266	7938	1000	1672	7438	7102
	**Patient 8**							
1	250	416	238	374	166	136	333	306
2	416	494	374	510	78	136	455	442
3	494	587	510	578	93	68	540.5	544
4	587	697	578	782	110	204	642	680
5	697	828	782	850	131	68	762.5	816
6	828	983	850	918	155	68	905.5	884
7	983	1168	918	1121	185	203	1075.5	1019.5
8	1168	1387	1121	1393	219	272	1277.5	1257
9	1387	1648	1393	1529	261	136	1517.5	1461
10	1648	1958	1529	2005	310	476	1803	1767
11	1958	2326	2005	2345	368	340	2142	2175
12	2326	2762	2345	2821	436	476	2544	2583
13	2762	3281	2821	3161	519	340	3021.5	2991
14	3281	3858	3161	5064	577	1903	3569.5	4112.5
15	3858	4630	5064	8054	772	2990	4244	6559
16	4630	8700	8054	8700	4070	646	6665	8377
	**Patient 9**							
1	250	416	238	374	166	136	333	306
2	416	494	374	510	78	136	455	442
3	494	587	510	578	93	68	540.5	544
4	587	697	578	646	110	68	642	612
5	697	828	646	782	131	136	762.5	714
6	828	983	782	850	155	68	905.5	816
7	983	1168	850	1054	185	204	1075.5	952
8	1168	1387	1054	1189	219	135	1277.5	1121.5
9	1387	1648	1189	1665	261	476	1517.5	1427
10	1648	1958	1665	1869	310	204	1803	1767
11	1958	2326	1869	2413	368	544	2142	2141
12	2326	2762	2413	2549	436	136	2544	2481
13	2762	3281	2549	3161	519	612	3021.5	2855
14	3281	3858	3161	5268	577	2107	3569.5	4214.5
15	3858	4630	5268	8054	772	2786	4244	6661
16	4630	8700	8054	8700	4070	646	6665	8377
	**Patient 10**							
1	188	313	188	354	125	166	250.5	271
2	313	438	354	448	125	94	375.5	401
3	438	563	448	658	125	210	500.5	553
4	563	688	658	816	125	158	625.5	737
5	688	813	816	927	125	111	750.5	871.5
6	813	938	927	1080	125	153	875.5	1003.5
7	938	1063	1080	1158	125	78	1000.5	1119
8	-	-	-	-	-	-	-	-
9	1068	1188	1158	1220	120	62	1128	1189
10	1188	1438	1220	1480	250	260	1313	1350
11	1438	1688	1480	1775	250	295	1563	1627.5
12	1688	1938	1775	2160	250	385	1813	1967.5
13	1938	2188	2160	2299	250	139	2063	2229.5
14	2188	2563	2299	2997	375	698	2375.5	2648
15	2563	2938	2997	3081	375	84	2750.5	3039
16	2938	3438	3081	4077	500	996	3188	3579
17	3438	3938	4077	4197	500	120	3688	4137
18	3938	4563	4197	4964	625	767	4250.5	4580.5
19	4563	5313	4964	5762	750	798	4938	5363
20	5313	6063	5762	6809	750	1047	5688	6285.5
21	6063	6938	6809	7702	875	893	6500.5	7255.5
22	6938	7938	7702	7938	1000	236	7438	7820
	**Patient 11**							
1	188	313	188	368	125	180	250.5	278
2	313	438	368	509	125	141	375.5	438,5
3	438	563	509	584	125	75	500.5	546.5
4	563	688	584	747	125	163	625.5	665.5
5	688	813	747	816	125	69	750.5	781.5
6	813	938	816	995	125	179	875.5	905.5
7	938	1063	995	1127	125	132	1000.5	1061
8	1063	1188	1127	1226	125	99	1125.5	1176.5
9	1188	1313	1226	1313	125	87	1250.5	1269.5
10	1313	1563	1313	1674	250	361	1438	1493.5
11	1563	1813	1674	1847	250	173	1688	1760.5
12	1813	2063	1847	2310	250	463	1938	2078.5
13	2063	2313	2310	2531	250	221	2188	2420.5
14	2313	2688	2531	3079	375	548	2500.5	2805
15	2688	3063	3079	3494	375	415	2875.5	3286.5
16	3063	3563	3494	4018	500	524	3313	3756
17	3563	4063	4018	4090	500	72	3813	4054
18	4063	4688	4090	4752	625	662	4375.5	4421
19	4688	5313	4752	5611	625	859	5000.5	5181.5
20	5313	6063	5611	6706	750	1095	5688	6158.5
21	6063	6938	6706	7846	875	1140	6500.5	7276
22	6938	7938	7846	7938	1000	92	7438	7892
	**Patient 12**							
1	70	170	70	170	100	100	120	120
2	170	300	170	325	130	155	235	247.5
3	300	469	325	487	169	162	384.5	406
4	469	690	487	702	221	215	579.5	594.5
5	690	982	702	1058	292	356	836	880
6	982	1368	1058	1453	386	395	1175	1255.5
7	1368	1881	1453	1919	513	466	1624.5	1686
8	1881	2564	1919	3076	683	1157	2222.5	2497.5
9	2564	3475	3076	3705	911	629	3019.5	3390.5
10	3475	4693	3705	4971	1218	1266	4084	4338
11	4693	6321	4971	6560	1628	1589	5507	5765.5
12	6321	8500	6560	8500	2179	1940	7410.5	7530
	**Patient 13**							
1	188	313	188	358	125	170	250.5	273
2	313	438	358	493	125	135	375.5	425.5
3	438	563	493	584	125	91	500.5	538.5
4	563	688	584	748	125	164	625.5	666
5	688	813	748	894	125	146	750.5	821
6	813	938	894	956	125	62	875.5	925
7	938	1063	856	1138	125	282	1000.5	997
8	1063	1188	1138	1318	125	180	1125.5	1228
9	1188	1313	1318	1541	125	223	1250.5	1429.5
10	1313	1563	1541	1808	250	267	1438	1674.5
11	1563	1813	1808	1870	250	62	1688	1839
12	1813	2063	1870	2373	250	503	1938	2121.5
13	2063	2313	2373	2490	250	117	2188	2431.5
14	2313	2688	2490	2793	375	303	2500.5	2641.5
15	2688	3063	2793	3216	375	423	2875.5	3004.5
16	3063	3563	3216	4002	500	786	3313	3609
17	3563	4063	4002	4447	500	445	3813	4224.5
18	4063	4688	4447	5017	625	570	4375.5	4732
19	4688	5313	5017	6196	625	1179	5000.5	5606.5
20	5313	6063	6196	6827	750	631	5688	6511.5
21	6063	6938	6827	7654	875	827	6500.5	7240.5
22	6938	7938	7654	7938	1000	284	7438	7796
	**Patient 14**							
1	188	313	188	315	125	127	250.5	251.5
2	313	438	315	512	125	197	375.5	413.5
3	438	563	512	688	125	176	500.5	600
4	563	688	688	761	125	73	625.5	724.5
5	688	813	761	936	125	175	750.5	848.5
6	813	938	936	1077	125	141	875.5	1006.5
7	938	1063	1077	1146	125	69	1000.5	1111.5
8	1063	1188	1146	1296	125	150	1125.5	1221
9	1188	1313	1296	1358	125	62	1250.5	1327
10	1313	1563	1358	1762	250	404	1438	1560
11	1563	1813	1762	1824	250	62	1688	1793
12	1813	2063	1824	2177	250	353	1938	2000.5
13	2063	2313	2177	2334	250	157	2188	2255.5
14	2313	2688	2334	2740	375	406	2500.5	2537
15	2688	3063	2740	3567	375	827	2875.5	3153.5
16	3063	3563	3567	4058	500	491	3313	3812.5
17	3563	4063	4058	4320	500	262	3813	4189
18	4063	4688	4320	5349	625	1029	4375.5	4834.5
19	4688	5313	5349	5579	625	230	5000.5	5464
20	5313	6063	5579	6595	750	1016	5688	6087
21	6063	6938	6595	7467	875	872	6500.5	7031
22	6938	7938	7467	7938	1000	471	7438	7702.5
	**Patient 15**							
1	70	170	70	197	100	127	120	133.5
2	170	300	197	354	130	157	235	275,5
3	300	469	354	480	169	126	384.5	417
4	469	690	480	816	221	336	579.5	648
5	690	982	816	1105	292	289	836	960.5
6	982	1368	1105	1394	386	289	1175	1249.5
7	1368	1881	1394	1985	513	591	1624.5	1689.5
8	1881	2564	1985	2805	683	820	2222.5	2395
9	2564	3475	2805	3965	911	1160	3019.5	3385
10	3475	4693	3965	7722	1218	3757	4084	5843.5
11	4693	6321	4722	6671	1628	1949	5507	5696.5
12	6321	8500	6671	8500	2179	1829	7410.5	7585.5
	**Patient 16**							
1	100	221	100	244	121	144	160.5	172
2	221	386	244	409	165	165	303.5	326.5
3	386	615	409	728	229	319	500.5	568.5
4	615	935	728	1015	320	287	775	871.5
5	935	1383	1015	1450	448	435	1159	1232.5
6	1383	2014	1450	2284	631	834	1698.5	1867
7	2014	2906	2284	3475	892	1191	2460	2879.5
8	2906	4169	3475	4569	1263	1094	3537.5	4022
9	4169	5959	4569	6309	1790	1740	5064	5439
10	5959	8500	6312	8500	2541	2188	7229.5	7406
11	-	-	-	-	-	-	-	-
12	-	-	-	-	-	-	-	-
	**Patient 17**							
1	188	313	188	337	125	149	250.5	262.5
2	313	438	337	501	125	164	375.5	419
3	438	563	501	563	125	62	500.5	532
4	563	688	563	751	125	188	625.5	657
5	688	813	751	882	125	131	750.5	816.5
6	813	938	882	1024	125	142	875.5	953
7	938	1063	1024	1227	125	203	1000.5	1125.5
8	-	-	-	-	-	-	-	-
9	1063	1313	1227	1397	250	170	1188	1312
10	1313	1563	1397	1808	250	411	1438	1602.5
11	1563	1813	1808	1984	250	176	1688	1896
12	1813	2188	1984	2203	375	219	2000.5	2093.5
13	2188	2563	2203	2653	375	450	2375.5	2428
14	2563	3063	2653	3505	500	852	2813	3079
15	3063	3563	3505	3900	500	395	3313	3702.5
16	3563	4188	3900	4315	625	415	3875.5	4107.5
17	4188	4938	4315	5275	750	960	4563	4795
18	4938	5813	5275	6519	875	1244	5375.5	5897
19	5813	6813	6519	7074	1000	555	6313	6796.5
20	6813	7938	7074	7938	1125	864	7375.5	7506
21	-	-	-	-	-	-	-	-
22	-	-	-	-	-	-	-	-
	**Patient 18**							
1	70	170	70	200	100	130	120	135
2	170	300	200	352	130	152	235	276
3	300	469	352	545	169	193	384.5	448.5
4	469	690	545	725	221	180	579.5	635
5	690	982	725	1098	292	373	836	911.5
6	982	1368	1098	1374	386	276	1175	1236
7	1368	1881	1374	2040	513	666	1624.5	1707
8	1881	2564	2040	2724	683	684	2222.5	2382
9	2564	3475	2724	3587	911	863	3019.5	3155.5
10	3475	4693	3587	4860	1218	1273	4084	4223.5
11	4693	6321	4869	6855	1628	1986	5507	5862
12	6321	8500	6855	8500	2179	1645	7410.5	7677.5
	**Patient 19**							
1	100	198	100	236	98	136	149	168
2	198	325	236	387	127	151	261.5	311.5
3	325	491	387	538	166	151	408	462.5
4	491	710	538	823	219	285	600.5	680.5
5	710	999	823	1147	289	324	854.5	985
6	999	1383	1147	1470	384	323	1191	1308.5
7	1383	1893	1470	2141	510	671	1638	1805.5
8	1893	2754	2141	2662	861	521	2323.5	2401.5
9	2574	3483	2662	3975	909	1313	3028.5	3318.5
10	3483	4698	3975	4727	1215	752	4090.5	4351
11	4698	6323	4727	7328	1625	2601	5510.5	6027.5
12	6323	8500	7328	8500	2177	1172	7411.5	7914
	**Patient 20**							
1	188	313	188	313	125	125	250.5	250.5
2	313	438	313	454	125	141	375.5	383.5
3	438	563	454	665	125	211	500.5	559.5
4	563	688	665	814	125	149	625.5	739.5
5	688	813	814	876	125	62	750.5	845
6	813	938	876	958	125	82	875.5	917
7	938	1063	958	1117	125	159	1000.5	1037.5
8	1063	1188	1117	1285	125	168	1125.5	1201
9	1188	1438	1285	1609	250	324	1313	1447
10	1438	1688	1609	1776	250	167	1563	1692.5
11	1688	1938	1776	2167	250	391	1813	1971.5
12	1938	2313	2167	2276	375	109	2125.5	2221.5
13	2313	2688	2276	2742	375	466	2500.5	2509
14	2688	3188	2742	2842	500	100	2938	2792
15	3188	3688	2842	2904	500	62	3438	2873
16	3686	4313	2904	4588	627	1684	3999.5	3746
17	4313	5063	4588	5492	750	904	4688	5040
18	5063	5938	5492	6541	875	1049	5500.5	6016.5
19	5938	6938	6541	7117	1000	576	6438	6829
20	6938	7935	7117	7938	997	821	7436.5	7527.5
21	-	-	-	-	-	-	-	-
22	-	-	-	-	-	-	-	-
	**Patient 21**							
1	188	313	188	313	125	125	250.5	250.5
2	313	438	313	447	125	134	375.5	380
3	438	563	447	578	125	131	500.5	512.5
4	563	688	578	781	125	203	625.5	679.5
5	688	813	781	893	125	112	750.5	837
6	813	938	893	973	125	80	875.5	933
7	938	1063	973	1161	125	188	1000.5	1067
8	1063	1188	1161	1223	125	62	1125.5	1192
9	1188	1313	1223	1327	125	104	1250.5	1275
10	1313	1563	1327	1829	250	502	1438	1578
11	1563	1813	1829	2016	250	187	1688	1922.5
12	1813	2063	2016	2446	250	430	1938	2231
13	2063	2313	2446	2599	250	153	2188	2522.5
14	2313	2688	2599	3001	375	402	2500.5	2800
15	2688	3063	3001	3562	375	561	2875.5	3281.5
16	3063	3563	3562	4189	500	627	3313	3875.5
17	3563	4063	4189	4688	500	499	3813	4438.5
18	4063	4688	4688	4866	625	178	4375.5	4777
19	4688	5313	4866	6232	625	1366	5000.5	5549
20	5313	6063	6232	6806	750	574	5688	6519
21	6063	6938	6806	7876	875	1070	6500.5	7341
22	6938	7938	7876	7938	1000	62	7438	7907
	**Patient 22**							
1	70	170	70	184	100	114	120	127
2	170	300	184	310	130	126	235	247
3	300	469	310	493	169	183	384.5	401.5
4	469	690	493	692	221	199	579.5	592.5
5	690	982	692	1163	292	471	836	927.5
6	982	1368	1163	1547	386	384	1175	1355
7	1368	1881	1547	2231	513	684	1624.5	1889
8	1881	2564	2231	2647	683	416	2222.5	2439
9	2564	3475	2647	4115	911	1468	3019.5	3381
10	3475	4693	4115	5439	1218	1324	4084	4777
11	4693	6321	5439	7050	1628	1611	5507	6244.5
12	6321	8500	7050	8500	2179	1450	7410.5	7775
	**Patient 23**							
1	188	313	188	361	125	173	250.5	274.5
2	313	438	361	507	125	146	375.5	434
3	438	563	507	584	125	77	500.5	545.5
4	563	688	584	755	125	171	625.5	669.5
5	688	813	755	885	125	130	750.5	820
6	813	938	885	1059	125	174	875.5	972
7	938	1063	1059	1214	125	155	1000.5	1136.5
8	1063	1188	1214	1310	125	96	1125.5	1262
9	1188	1313	1310	1372	125	62	1250.5	1341
10	1313	1563	1372	1738	250	366	1438	1555
11	1563	1813	1738	2050	250	312	1688	1894
12	1813	2063	2050	2130	250	80	1938	2090
13	2063	2313	2130	2368	250	238	2188	2249
14	2313	2688	2368	2956	375	588	2500.5	2662
15	2688	3063	2956	3676	375	720	2875.5	3316
16	3063	3563	3676	3805	500	129	3313	3740.5
17	3563	4063	3805	4538	500	733	3813	4171.5
18	4063	4688	4538	4975	625	437	4375.5	4756.5
19	4688	5313	4975	6196	625	1221	5000.5	5585.5
20	5313	6063	6196	6827	750	631	5688	6511.5
21	6063	6938	6827	7654	875	827	6500.5	7240.5
22	6938	7938	7654	7938	1000	284	7438	7796
	**Patient 24**							
1	188	313	188	315	125	127	250.5	251.5
2	313	438	315	517	125	202	375.5	416
3	438	563	517	641	125	124	500.5	579
4	563	688	641	781	125	140	625.5	711
5	688	813	781	942	125	161	750.5	861.5
6	813	938	942	1077	125	135	875.5	1009.5
7	938	1063	1077	1193	125	116	1000.5	1135
8	1063	1188	1193	1296	125	103	1125.5	1244.5
9	1188	1313	1296	1358	125	62	1250.5	1327
10	1313	1563	1358	1764	250	406	1438	1561
11	1563	1813	1764	1826	250	62	1688	1795
12	1813	2063	1826	2190	250	364	1938	2008
13	2063	2313	2190	2355	250	165	2188	2272.5
14	2313	2688	2355	2740	375	385	2500.5	2547.5
15	2688	3063	2740	3567	375	827	2875.5	3153.5
16	3063	3563	3567	4022	500	455	3313	3794.5
17	3563	4063	4022	4288	500	266	3813	4155
18	4063	4688	4288	5349	625	1061	4375.5	4818.5
19	4688	5313	5349	5579	625	230	5000.5	5464
20	5313	6063	5579	6537	750	958	5688	6058
21	6063	6938	6537	7467	875	930	6500.5	7002
22	6938	7938	7467	7938	1000	471	7438	7702.5
	**Patient 25**							
1	100	198	100	219	98	119	149	159.5
2	198	325	219	341	127	122	261.5	280
3	325	491	341	519	166	178	408	430
4	491	710	519	797	219	278	600.5	658
5	710	999	797	1052	289	255	854.5	924.5
6	999	1383	1052	1611	384	559	1191	1331.5
7	1383	1893	1611	2265	510	654	1638	1938
8	1893	2754	2265	2888	861	623	2323.5	2576.5
9	2574	3483	2888	3452	909	564	3028.5	3170
10	3483	4698	3452	5197	1215	1745	4090.5	4324.5
11	4698	6323	5197	7158	1625	1961	5510.5	6177.5
12	6323	8500	7158	8500	2177	1342	7411.5	7829
	**Patient 26**							
1	100	208	100	237	108	137	154	168.5
2	208	352	237	354	144	117	280	295.5
3	352	545	354	637	193	283	448.5	495.5
4	545	806	637	956	261	319	675.5	796.5
5	806	1160	956	1317	354	361	983	1136.5
6	1160	1643	1317	1891	483	574	1401.5	1604
7	1643	2303	1891	2644	660	753	1973	2267.5
8	2303	3208	2644	3459	905	815	2755.5	3051.5
9	3208	4450	3459	5033	1242	1574	3829	4246
10	4450	6155	5033	6365	1705	1332	5302.5	5699
11	6155	8500	6365	8500	2345	2135	7327.5	7432.5
12	-	-	-	-	-	-	-	-
	**Patient 27**							
1	188	313	188	328	125	140	250.5	258
2	313	438	328	490	125	162	375.5	409
3	438	563	490	599	125	109	500.5	544.5
4	563	813	599	927	250	328	688	763
5	813	1063	927	1176	250	249	938	1051.5
6	1063	1313	1176	1321	250	145	1188	1248.5
7	1313	1563	1321	1584	250	263	1438	1452.5
8	1563	1813	1584	1964	250	380	1688	1774
9	1813	2188	1964	2410	375	446	2000.5	2187
10	2188	2563	2410	2898	375	488	2375.5	2654
11	2563	3063	2898	3312	500	414	2813	3105
12	3063	3563	3312	4147	500	835	3313	3729.5
13	3563	4188	4147	4789	625	642	3875.5	4468
14	4188	4938	4789	5895	750	1106	4563	5342
15	4938	5813	5895	6430	875	535	5375.5	6162.5
16	5813	6813	6430	7256	1000	826	6313	6843
17	6813	7938	7256	7938	1125	682	7375.5	7597
18	-	-	-	-	-	-	-	-
19	-	-	-	-	-	-	-	-
20	-	-	-	-	-	-	-	-
21	-	-	-	-	-	-	-	-
22	-	-	-	-	-	-	-	-

## Appendix B

**Table A2:** The electrode position on post-operative CT scanner, center frequency deduced from the Greenwood map, default central frequency, and central frequency after fitting. BML (basilar membrane length) in mm, e# (electrode number), ERD (electrode to round window distance) in mm, GF (center frequency according to Greenwood equation) in Hz, DF (default center frequency) in Hz, EAF (Evolutionary Algorithm center frequency). -: deactivated electrodes.

ID	CI Brand/Array	BML	e#	ERD	GF	DF	EAF
1	MEDEL/Flex 28	30.7	1	1.56	16,140.9	7327.5	7288
			2	3.53	11,796.2	5302.5	5072.5
			3	5.67	8379.27	3829	3558.5
			4	7.76	5988.13	2755.5	2482.5
			5	9.73	4351.89	1973	1768
			6	11.78	3110.85	1401.5	1301.5
			7	13.97	2160.84	983	819.5
			8	16.04	1519.15	675.5	569.5
			9	18.27	1026.09	448.5	408
			10	20.3	705.461	280	263.5
			11	22.41	464.833	154	147.5
2	MEDEL/Flex 28	30.7	1	2.37	14,276.1	7327.5	6817
			2	4.42	10,350.7	5302.5	4638.5
			3	6.55	7399.64	3829	3477
			4	8.45	5475.08	2755.5	2497.5
			5	10.5	3945.21	1973	1873.5
			6	12.5	2854.92	1401.5	1324
			7	14.71	1984.74	983	940
			8	16.67	1426.68	675.5	661.5
			9	18.51	1036.59	448.5	406
			10	20.47	726.911	280	238.5
			11	22.32	509.428	154	146.5
3	COCHLEAR/422	26.6	1	3.26	11,366.9	7438	7199
			2	4.29	9401.1	6500.5	6209.5
			3	5.09	8109	5688	5525.5
			4	5.97	6888.73	5000.5	4850
			5	6.85	5848.85	4375.5	4231.5
			6	7.6	5084.86	3813	3473.5
			7	8.4	4376.94	3313	3061
			8	9.21	3757.73	2875.5	2724
			9	10.02	3223.31	2500.5	2161.5
			10	10.94	2704.49	2188	1846
			11	11.76	2309.8	1938	1728
			12	12.59	1965.93	1688	1565.5
			13	13.53	1634.27	1438	1295.5
			14	14.39	1376.69	1250.5	1098
			15	15.06	1202.13	1125.5	1034
			16	15.8	1032.51	1000.5	936
			17	16.4	910.776	875.5	796.5
			18	17.16	744.471	750.5	669.5
			19	18.08	632.784	625.5	544
			20	18.75	543.532	500.5	440
			21	18.92	522.563	375.5	353.5
			22	18.11	628.511	250.5	243
5	MEDEL/Flex 28	34.2	1	2.02	15,503.9	7535	7188.5
			2	4.12	11,483.7	5824	5282
			3	6.34	8350.89	4501.5	4012
			4	8.63	6000.88	3479.5	2975.5
			5	10.47	4592.95	2689.5	2361.5
			6	12.46	3430.86	2078.5	1801
			7	14.17	2662.78	1606.5	1329.5
			8	16.75	1804.41	1242	1069.5
			9	18.91	1291.24	960	875
			10	20.97	928.2	742	701.5
			11	23.15	643.389	573.5	563
8	AB/HiFOCUS Mid-scala	28.6	1	2.22	14,160.8	6665	8377
			2	3.32	11,732.9	4264	6559
			3	4.1	10,265.3	3589.5	4112.5
			4	4.9	8948.25	3021.5	2991
			5	5.91	7520.72	2544	2583
			6	6.86	6383.19	2142	2175
			7	7.9	5330.47	1803	1767
			8	8.93	4455.28	1517.5	1461
			9	9.99	3700.4	1277.5	1257
			10	10.8	3208.18	1075.5	1019.5
			11	11.8	2686.5	905.5	884
			12	12.8	2245.98	762.5	816
			13	13.8	1873.97	642	680
			14	14.8	1559.83	540.5	544
			15	15.8	1294.56	455	442
			16	16.8	1070.55	333	306
9	AB/HiFOCUS Mid-scala	27.5	1	1.5	15,849.7	6665	8377
			2	2.5	13,270.6	4264	6661
			3	3.46	11,186.7	3589.5	4214.5
			4	4.46	9359.48	3021.5	2855
			5	5.5	7770.99	2544	2481
			6	6.5	6494.51	2142	2141
			7	7.56	5365.4	1803	1767
			8	8.32	4676.04	1517.5	1427
			9	9.26	3941.48	1277.5	1121.5
			10	10.44	3175.68	1075.5	952
			11	11.27	2724.68	905.5	816
			12	12.3	2249.21	762.5	714
			13	13.28	1870.15	642	612
			14	14.32	1533.28	540.5	544
			15	15.08	1323.28	455	442
			16	16.12	1077.81	333	306
11	COCHLEAR/CI522	26.8	1	4.22	9578.87	7438	7892
			2	4.45	9183.58	6500.5	7276
			3	5.83	7127.34	5688	6158.5
			4	6.35	6476.01	5000.5	5181.5
			5	6.78	5981.71	4375.5	4421
			6	9.29	3750.16	3813	4054
			7	9.88	3356.77	3313	3756
			8	10.8	2821.1	2875.5	3286.5
			9	12.4	2077.21	2500.5	2805
			10	13.4	1710.26	2188	2420.5
			11	14.4	1403.89	1938	2078.5
			12	15.2	1195.63	1688	1760.5
			13	16.2	974.22	1438	1493.5
			14	17.51	738.51	1250.5	1269.5
			15	18.12	646.37	1125.5	1176.5
			16	19.12	515.63	1000.5	1061
			17	19.9	428.83	875.5	905.5
			18	20.6	360.68	750.5	781.5
			19	20.12	406.48	625.5	665.5
			20	21.58	278.64	500.5	546.5
			21	22.2	233.74	375.5	438.5
			22	23.3	165.46	250.5	278
13	COCHLEAR/CI522	23.2	1	1.71	14,434.22	7438	7796
			2	2.42	12,428.74	6500.5	7240.5
			3	3.39	10,127.08	5688	6511.5
			4	4.17	8585.74	5000.5	5606.5
			5	4.96	7260.21	4375.5	4732
			6	5.6	6335.4	3813	4224.5
			7	6.44	5294.51	3313	3609
			8	7.19	4507.27	2875.5	3004.5
			9	8.12	3687.41	2500.5	2641.5
			10	9.15	2946.89	2188	2431.5
			11	10.1	2391.39	1938	2121.5
			12	11.2	1871.61	1688	1839
			13	12	1561.82	1438	1674.5
			14	13.1	1212,00	1250.5	1429.5
			15	14	979.81	1125.5	1228
			16	14.9	787.33	1000.5	997
			17	15.7	644.06	875.5	925
			18	16.7	495.5	750.5	821
			19	17.3	420.14	625.5	666
			20	18.2	323.39	500.5	538.5
			21	19.2	235.16	375.5	425.5
			22	20	176.69	250.5	273
14	COCHLEAR/CI522	29.2	1	6.84	6562.82	7438	7702.5
			2	7.92	5464.23	6500.5	7031
			3	8.86	4655.58	5688	6087
			4	9.64	4073.82	5000.5	5464
			5	10.7	3394.54	4375.5	4834.5
			6	11.7	2854.28	3813	4189
			7	12.2	2615.9	3313	3812.5
			8	13.1	2233.54	2875.5	3153.5
			9	14	1904.12	2500.5	2537
			10	14.7	1679.78	2188	2255.5
			11	15.6	1427.04	1938	2000.5
			12	16.7	1165.16	1688	1793
			13	17.8	946.89	1438	1560
			14	19	750.01	1250.5	1327
			15	20	613.33	1125.5	1221
			16	20.7	530.27	1000.5	1111.5
			17	21.7	427.13	875.5	1006.5
			18	22.6	347.84	750.5	848.5
			19	23.4	286.62	625.5	724.5
			20	24	245.74	500.5	600
			21	25.1	180.58	375.5	413.5
			22	25.9	140.11	250.5	251.5
15	MEDEL/Flex 28	28.45	1	0.75	18,184.98	7410.5	7585.5
			2	2.89	12,595.75	5507	5696.5
			3	4.85	8985.88	4084	5843.5
			4	7.07	6115.86	3019.5	3385
			5	9.24	4184.53	2222.5	2395
			6	11.2	2957.73	1624.5	1689.5
			7	13.6	1918.25	1175	1249.5
			8	16.4	1136.75	836	960.5
			9	18.2	798.78	579.5	648
			10	20.4	504.18	384.5	417
			11	22.5	309.15	235	275.5
			12	24.4	183.66	120	133.5
16	MEDEL/Flex 28	28.2	1	0.87	17,791.38	7229.5	7406
			2	2.39	13,675.99	5064	5439
			3	4.68	9187.52	3537.5	4022
			4	6.82	6320.85	2460	2879.5
			5	8.7	4538.94	1698.5	1867
			6	11	3012.26	1159	1232.5
			7	13.2	2019.94	775	871.5
			8	15.4	1339.44	500.5	568.5
			9	18	805.29	303.5	326.5
			10	20.2	506.49	160.5	172
			11	22.3	309.32	-	-
			12	24.2	182.85	-	-
17	COCHLEAR/CI522	24.8	1	2.64	12,299.22	-	-
			2	3.52	10,337.09	-	-
			3	4.33	8805.66	7375.5	7506
			4	5.19	7423.81	6313	6796.5
			5	6.11	6180.84	5375.5	5897
			6	6.91	5267.15	4563	4795
			7	7.78	4422.64	3875.5	4107.5
			8	8.67	3694.89	3313	3702.5
			9	9.77	2953.55	2813	3079
			10	10.8	2389.68	2375.5	2428
			11	11.6	2023.53	2000.5	2093.5
			12	12.6	1639.29	1688	1896
			13	13.5	1352.02	1438	1602.5
			14	14.5	1086.73	1188	1312
			15	15.4	888.4	-	-
			16	16.5	688.81	1000.5	1125.5
			17	17.3	568.31	875.5	953
			18	18.1	465.21	750.5	816.5
			19	18.8	387.29	625.5	657
			20	19.6	310.33	500.5	532
			21	20.4	244.49	375.5	419
			22	21.2	188.16	250.5	262.5
21	COCHLEAR/CI522	28.1	1	5.48	7964.02	7438	7907
			2	6.24	6969.87	6500.5	7341
			3	7.33	5752.95	5688	6519
			4	8.27	4872.01	5000.5	5549
			5	9.34	4028.21	4375.5	4777
			6	10.5	3272.96	3813	4438.5
			7	11.3	2833.31	3313	3875.5
			8	12.5	2277.55	2875.5	3281.5
			9	13.6	1859.68	2500.5	2800
			10	14.5	1571.98	2188	2522.5
			11	15.4	1325.56	1938	2231
			12	16.3	1114.49	1688	1922.5
			13	17.4	897.19	1438	1578
			14	18.2	763.09	1250.5	1275
			15	19	646.23	1125.5	1192
			16	19.8	544.4	1000.5	1067
			17	20.7	445.41	875.5	933
			18	21.6	360.62	750.5	837
			19	22.7	273.33	625.5	679.5
			20	23.2	238.79	500.5	512.5
			21	24.1	183.65	375.5	380
			22	24.9	141.31	250.5	250.5
24	COCHLEAR/CI522	24.3	1	1.33	15,835.2	7438	7702.5
			2	2.47	12,591.78	6500.5	7002
			3	3.28	10,695.68	5688	6058
			4	4.15	8972.32	5000.5	5464
			5	4.84	7802.55	4375.5	4818.5
			6	5.65	6619.38	3813	4155
			7	6.53	5532.69	3313	3794.5
			8	7.43	4601.63	2875.5	3153.5
			9	8.44	3737.31	2500.5	2547.5
			10	9.52	2986.43	2188	2272.5
			11	10.3	2536.15	1938	2008
			12	11.4	2008.96	1688	1795
			13	12.2	1691.89	1438	1561
			14	13.3	1330.67	1250.5	1327
			15	14.1	1113.42	1125.5	1244.5
			16	15.2	865.92	1000.5	1135
			17	16	717.06	875.5	1009.5
			18	16.8	590.11	750.5	861.5
			19	17.7	469.49	625.5	711
			20	18.5	378.97	500.5	579
			21	19.4	292.97	375.5	416
			22	20.5	206.76	250.5	251.5
26	MEDEL/Flex 28	25.4	1	0	20,677.07	-	-
			2	2.19	13,578.18	7327.5	7432.5
			3	4.35	8951.27	5302.5	5699
			4	5.24	7533.49	3829	4246
			5	7.01	5336.82	2755.5	3051.5
			6	9.25	3433.53	1973	2267.5
			7	10.5	2675.59	1401.5	1604
			8	12.1	1934.82	983	1136.5
			9	14	1303.4	675.5	796.5
			10	16	844.59	448.5	495.5
			11	18.3	493.51	280	295.5
			12	20.3	289.49	154	168.5
